# Smokeless tobacco consumption impedes metabolic, cellular, apoptotic and systemic stress pattern: A study on Government employees in Kolkata, India

**DOI:** 10.1038/srep18284

**Published:** 2015-12-16

**Authors:** Sushobhan Biswas, Krishnendu Manna, Ujjal Das, Amitava Khan, Anirban Pradhan, Aaveri Sengupta, Surajit Bose, Saurabh Ghosh, Sanjit Dey

**Affiliations:** 1 Department of Physiology, University of Calcutta, 92, A.P.C Road, Kolkata 700009, West Bengal, India; 2 Human Genetics Unit, Indian Statistical Institute, 203 B.T. Road, Kolkata 700 108, West Bengal, India

## Abstract

Smokeless tobacco (SLT) remains a threat amongst a large population across the globe and particularly in India. The oral use of tobacco has been implicated to cause physiological stress leading to extreme toxicological challenge. The study included 47 SLT-users and 44 non-users providing a spectrum of pathophysiological, clinico-biochemical, antioxidant parameters, cell cycle progression study of PBMC and morphological changes of red blood cells (RBC). The expressions of p53, p21, Bax, Bcl-2, IL-6, TNF- α, Cox-2, iNOS were analyzed from thirteen representative SLT-users and twelve non-users. Difference in CRP, random glucose, serum cholesterol, TG, HLDL-C, LDL-C, VLDL-C, neutrophil count, monocyte count, ESR, SOD (PBMC) and TBARS (RBC membrane) were found to be statistically significant (p < 0.05) between the studied groups. The current study confers crucial insight into SLT mediated effects on systemic toxicity and stress. This has challenged the metabolic condition leading to a rise in the inflammatory status, increased apoptosis and RBC membrane damage. The above findings were substantiated with metabolic, clinical and biochemical parameters. This is possibly the first ever in-depth report and remains an invaluable document on the fatal effects of SLT.

The habit of oral use of smokeless tobacco (SLT), such as “chewing tobacco” or “spit tobacco” is becoming a global threat to human health with every passing day due to its numerous deleterious effects. The use of SLT was reported to be more prevalent in the South Asian countries in comparison to the Western world^1^, although current studies have revealed facts about its worldwide usage^2^. Chewing tobacco or spit tobacco, which is largely used in India and partly across the global population, is mixed with betel leaves, areca nut, lime and catechu. It is available in common Indian market as “*gutkha*”^3^ and comprises of a vast number of compounds, of which many are toxicants and carcinogens, that are also found in cigarette smoke (CS) causing a plethora of health diseases^4^. The tobacco-specific nitrosamines (TSNAs) are considered to be the most potent among the 28 known carcinogens in smokeless tobacco (National Cancer Institute, 1992), due to its strong carcinogenicity^5^. Besides the toxic chemicals like polycyclic aromatic hydrocarbons, nitrate, nitrite, nicotine, acrolein, chemicals such as crotonaldehyde, substantial amounts of formaldehyde, acetaldehyde, etc have also been reported to be present in the smokeless tobacco^6^,^7^. There have been a number of reports regarding SLT- induced cytotoxicity^8^,^9^,^10^, although the exact molecular mechanism remains unknown. Exposure to SLT is known to cause cell death and apoptosis in several cultured cell lines including oral keratinocytes^8^,^11^ and macrophages^12^.

The mixture of SLT is chewed slowly which ultimately forms an aqueous extract of SLT in saliva. This extract is available to be absorbed locally and often ingested making its way into the systemic circulation. Keeping SLT in mouth, either as snuff or chewing tobacco is identified to enhance wrinkling in the oral mucosa. It is responsible to create oral injury and inflammation which give rise to snuff dipper’s lesion, commonly referred to as leukoplakia. This is characterized by an increased occurrence of gingival recession with associated loss of attachment, cervical abrasion and oral tissue damage^1^. It has been reported that oral administration of the aqueous extract of smokeless tobacco in male rats resulted in the apoptosis and damage of lung, liver and kidney tissues, along with significant up-regulation of pro-apoptotic and inflammatory genes^12^.

SLT consumption is more prevalent among lower socio-economic groups in India that includes the class of the poor, semi-skilled manual workers, unemployed people with meagre education. It is believed that the usage of SLT relieves the work related stress among its users. It is supposed to have healing properties for curing toothache, headache and stomach ache which dictates many adults to accede to its usage. Some parents even encourage their children to use SLT^13^. Curiosity, peer pressure, offered by friends and acquaintances contribute to the initiation of its use^14^.

There are evidences which support that SLT has a strong association with a range of oral cavity lesions, of which oral cancer is the most prominent, including leukoplakia, smokeless tobacco keratosis, submucous fibrosis, leukoedema, hairy tongue, tooth decay etc. Consumption of SLT may be a contributory factor in the expansion of cardiovascular disease, peripheral vascular disease, hypertension, diabetes, hypercholesterolemia, peptic ulcers and, foetal morbidity and mortality^13^,^15^. Compared to the non-users, increased frequency of cardiovascular, respiratory and renal diseases has been observed in SLT-users, as revealed by epidemiological studies^15^,^16^. Nearly six million people die each year as a consequence of tobacco use accounting for 12% of worldwide adult mortality^17^. Continuation of the existing tobacco use pattern is projected to cause around 10 million deaths each year by 2020^18^. With the course of events and actions, SLT and CS both seem to be equally powerful in altering the cellular and metabolic changes. Gajalakshmi *et al.* (2012), Giovino (2012) and Sinha *et al.* (2012) have delineated the association between SLT use and human mortality^19^,^20^,^21^.

In this study, we investigated with the objective of analyzing effects of chewing tobacco among the northern sub-population of Kolkata, India. Since the adverse effects of SLT among users are well known, we were interested to perform one-tailed tests of hypothesis with respect to different clinical parameters. A spectrum of different physiological parameters was studied between the SLT-user and non-user groups with a mechanistic view point.

Measurements of total haematological and some clinico-biochemical parameters were done. Antioxidant assays were performed from isolated peripheral blood mononuclear cells (PBMC) and red blood cell (RBC) membranes to measure the extent of challenge to normal metabolic pattern leading to oxidative stress. Cell cycle analysis of PBMC was performed using flowcytometer to detect the distribution of DNA content in different phases of cell cycle. Level of different pro- and anti- apoptotic markers such as p53, p21, Bax, Bcl-2 and pro-inflammatory cytokines such as IL-6, TNF-α, iNOS, Cox-2 were analysed. Structural changes of RBC were analysed microscopically among the studied groups. We reported that SLT use aggravated the systemic stress and inflammatory developments; moreover, it grossly perturbed the metabolic harmony as reflected in apoptotic developments and RBC morphology change. This is perhaps the first exhaustive report on the menace of SLT on Indian population to make a road map for the therapeutic approach of the disease and open further avenues for research in this field.

## Results

### Clinical parameters

In Table 1 we presented the clinical parameters. We found that, SLT-user group had higher levels of c-reactive protein (CRP), glucose (random), urea, serum cholesterol, serum triglyceride (TG), high-density lipoprotein-cholesterol (HDL-C), low-density lipoprotein-cholesterol (LDL-C), very low-density lipoprotein-cholesterol (VLDL-C), leukocyte count, neutrophil count, platelet count, erythrocyte sedimentation rate (ESR), mean corpuscular haemoglobin (MCH) and mean corpuscular haemoglobin concentration (MCHC) level compared to the non-user group. Regression analysis showed that the values of CRP, random glucose, serum cholesterol, TG, HLDL-C, LDL-C, VLDL-C, neutrophil count, monocyte count, ESR varied between the studied groups and were statistically significant (p < 0.05). From the summary statistics in Table 1 it was observed that the values of haemoglobin, erythrocyte count, lymphocyte count, monocyte count, eosinophil count, packed cell volume (PCV), mean corpuscular volume (MCV) were higher in the non-user group, however the differences were not statistically significant (p > 0.05, from regression analysis) except monocyte count (p < 0.05). A lower value was observed for urea, creatinine in the non-user group and the mean difference was not statistically significant (p > 0.05).

### Chewers have shown compromised antioxidant status for PBMC and RBC membrane

The purpose of determination of antioxidant parameters was to evaluate the antioxidant defence system after SLT chewing. We examined the structural integrity of RBC in SLT-user and non-user individuals by measuring the concentrations of thiobarbituric acid reactive substance (TBARS) and antioxidant status from RBC membrane. From Tables 2 and 3, we found that SLT-user group showed higher level (3.70 ± 0.51 nmoles/mg of protein and 15.16 ± 11.73 μmoles/mg of protein) of malondialdehyde (MDA) formation which augmented higher amount of TBARS as compared to non-user group (3.56 ± 0.40 nmoles/mg of protein and 8.14 ± 9.71 μmoles/mg of protein) from PBMC and RBC membrane respectively. The mean difference of TBARS levels for RBC membrane was statistically significant (p < 0.05). A lower value of reduced glutathione (GSH) level was observed in the chewers group (42.72 ± 26.40 and 366.47 ± 137.98 nmoles/mg of protein) as compared to the non-chewers group (53.25 ± 28.03 and 415.41 ± 173.71 nmoles/mg of protein) for PBMC and RBC membrane respectively (Tables 2 and 3). Difference of superoxide dismutase (SOD) activity observed was not statistically significant between the studied groups for both PBMC (1.26 ± 0.34 and 1.1 ± 0.33 U/g of protein for SLT-users and non-users respectively) and RBC membrane (1.26 ± 0.37 and 1.04 ± 0.26 U/g of protein for SLT-users and non-users respectively). Similarly, the difference of catalase activity was not statistically significant between the studied groups for both PBMC (2.08 ± 0.56 and 2.10 ± 0.55 mmoles of H_2_O_2_ reduced/mg of protein for SLT-users and non-users respectively) and RBC membrane (1.75 ± 0.49 and 1.55 ± 0.57 of H_2_O_2_ reduced/mg of protein for SLT-users and non-users respectively) (Tables 2 and 3).

### SLT alters RBC morphology

**Figure 1:**
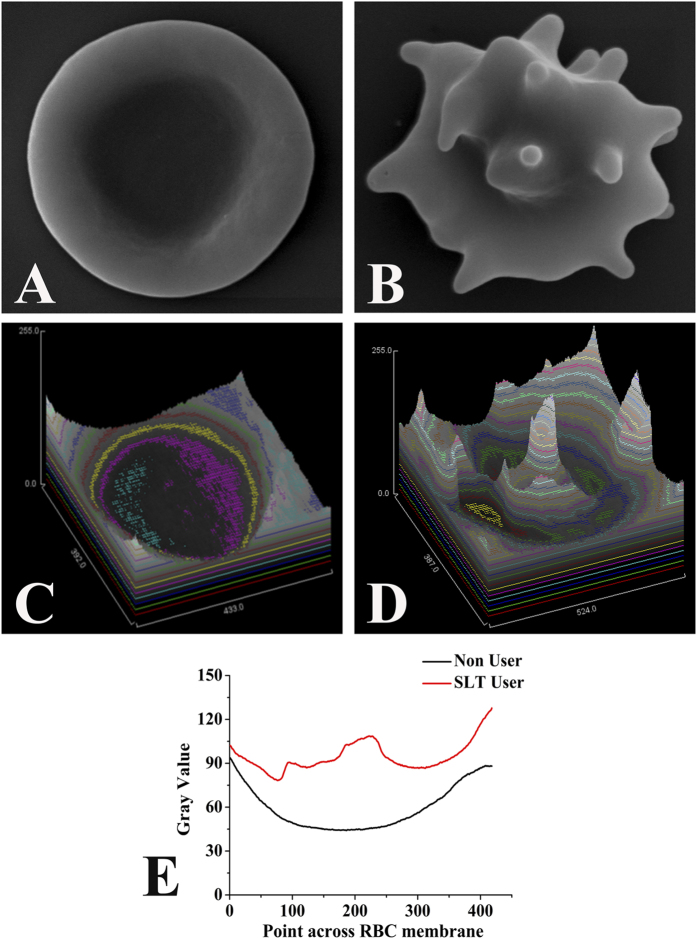
Red blood cell (RBC) ultra structure of the SLT-user and non-user. Scanning electron microscopy images of RBC from (**A**) Non-user individual. Scale-1 μm, magnification- 25,000 × machine magnification, (**B**) SLT-chewer individual. Scale-1 μm, magnification-30,000 × machine magnification. (**C,D**) Surface topology plot of RBC from non-user and SLT-user individuals respectively. (**E**) Quantitative representation of RBC surface topology showing alteration in SLT-user (red line) compared to non-user (black line).

Fig. 1A showed the image of a typical erythrocyte (RBC) collected from a non-user (at 25000 × machine magnification) and 1B represented RBC micrograph of a representative SLT-user (at 30000 × machine magnification). The shape of healthy RBCs had usual architecture, whereas a general shape alteration in SLT-user was seen with the erythrocyte deforming from its typical discoid appearance to form multi-pointed extensions. The surface topologies were analysed and represented in Fig. 1C–E.

### Analysis of cell cycle progression

**Figure 2:**
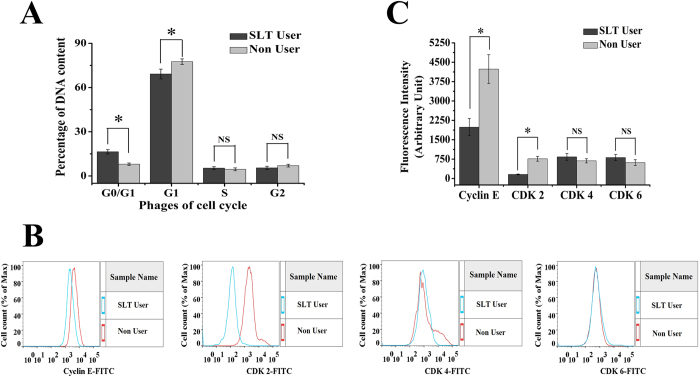
Cell cycle dynamics of peripheral blood mononuclear cells from SLT-user and non-user groups. (**A**) SLT induced increase in DNA content of G0/G1 phase and decrease in DNA content of G1 phase in SLT-user group (dark gray) compared to non-user group (light gray), SLT-user: N = 47; non-user: N = 44. (**B,C**) Cell cycle transition regulatory proteins were modulated in the course of cell cycle arrest, (**B**) Representative histogram showing variable expressions of different cell cycle regulatory proteins like cyclin-E, CDK- 2, CDK-4, CDK-6 by FITC-fluorescence intensity. (**C**) Quantitative representation of cyclin-E, CDK-2, CDK-4, CDK-6 fluorescence intensity values obtained from flow cytometric analysis between the SLT-user (dark gray) and non-user (light gray) group, SLT-user: N = 10; non-user: N = 10. Data are mean  ±  SEM. *p < 0.05 significant difference between the two test groups, NS: not significant.

In this present study, a correlation of apoptosis with the regulation of cell cycle progression was investigated. The proportion of DNA content in G0/G1, G1, S, G2 phases in the SLT-user and non-user groups were determined using flowcytometer after propidium iodide (PI) staining. As shown in the Fig. 2A, SLT-user group had increased percentage of DNA content in the G0/G1 phase of cell cycle. The percentages of DNA content in the G0/G1 phase were 16.41 ± 1.48 and 7.97 ± 0.81 (p < 0.05) for SLT-user and non-user groups respectively. Notably, the percentage of DNA content in G1 phase decreased (p < 0.05) in the SLT-user group and it remain unaltered in S and G2 phases between the studied groups (Fig. 2A). It is reported that CDK-2-cyclin-E complex is involved in regulating the transition from G1 to S phase^22^. Thus, expressions of CDK-2 and cyclin-E were determined between the studied groups. As shown in Fig. 2C, there were dramatic down-regulations of CDK-2 and cyclin-E in SLT-user group as compared to the non-user group. The activity of CDK-4 and CDK-6 were restricted to the G1-S phase, we found the difference of CDK-4 and CDK-6 were not statistically significant between the studied groups (Fig. 2B,C).

### p53 and p21 levels were higher in SLT-user group

**Figure 3:**
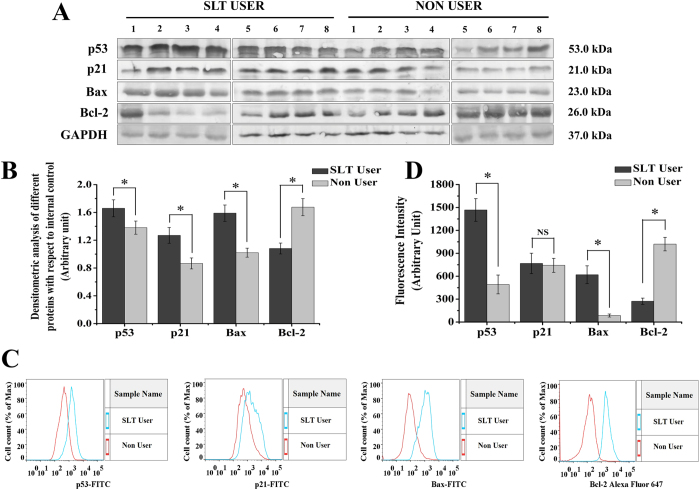
SLT induced cell cycle arrest and induction of apoptotic markers like p53, p21, Bax, Bcl-2. (**A**) Representative immunblots of eight SLT-users and eight non-users. (**B**) Quantitative analysis of densitometric values obtained from immunblot analysis showing increased expression of p53, p21, Bax and decreased expression of Bcl-2 in the SLT-user group (dark gray) compared to non-user group (light gray), SLT-user: N = 13 and non-user: N = 12. (**C**) Representative histogram showing variable expression of p53, p21, Bax by FITC-fluorescence intensity and Bcl-2 by Alexa Fluor 647- fluorescence intensity. (**D**) Quantitative representation of p53, p21, Bax, Bcl-2 fluorescence intensity values obtained from flow cytometric analysis between the SLT-user (dark gray) and non-user (light gray) group, SLT-user: N = 10 and non-user: N = 10. Data are mean  ±  SEM. *p < 0.05 significant difference between the two test groups, NS: not significant.

Densitometric analysis of immune blot data of the SLT-user group (thirteen SLT-users) and non-user group (twelve non-users) were represented in Fig. 3A,B. It showed significant higher level of expression for both p53 and p21in the SLT-user group (p < 0.05). Flowcytometry analysis showed SLT-user group (ten SLT-users) had significantly higher (p < 0.05) level of expression for p53 while the difference was not significant (p > 0.05) for p21 expression (Fig. 3D). Histogram representation of flowcytometry data of representative SLT-user and non-user were presented in Fig. 3C.

### Higher Bax and lower Bcl-2 levels were observed in SLT-user group

Densitometric analysis of Bax and Bcl-2 from the SLT-user group (thirteen SLT-users) and non-user group (twelve non-users) were represented in Fig. 3A,B. It showed significant higher level of expression for Bax in the SLT-user group (p < 0.05) and Bcl-2 in the non-user group (p < 0.05). Similar results were also found in flowcytometry analysis with ten SLT-user and ten non-user (Fig. 3D). Histograms from flowcytometry data of representative SLT-user and non-user were represented in Fig. 3C.

### IL-6, TNF-α, iNOS, Cox-2 levels were higher in SLT-user group

**Figure 4:**
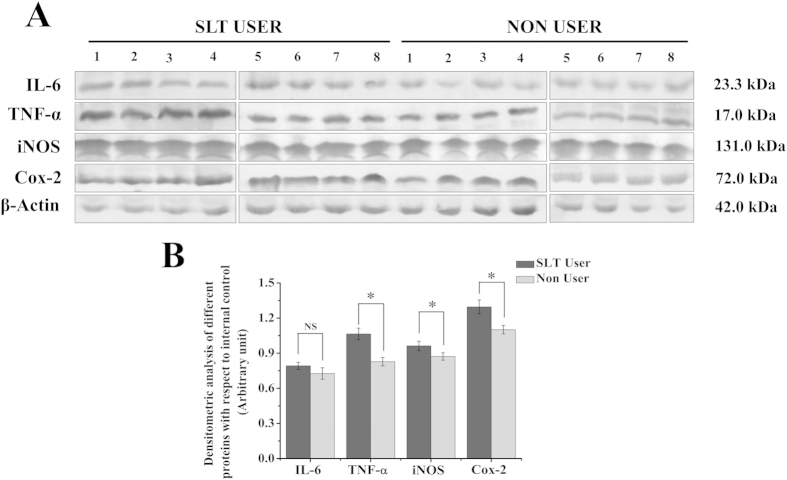
SLT induced enhancement of pro-inflammatory cytokines like IL-6, TNF-α, iNOS, Cox-2. (**A**) Representative immunblots of eight SLT-users and eight non-users. (**B**) Quantitative analysis of densitometric values obtained from immunblot analysis showing increased expression of IL-6, TNF-α, iNOS, Cox-2 in the SLT-user group (dark gray) compared to non-user group (light gray), SLT-user: N = 13 and non-user: N = 12. Data are mean  ±  SEM. *p < 0.05 significant difference between the two test groups, NS: not significant.

SLT-user group (thirteen SLT-users) showed significantly higher (p < 0.05) level of expression for pro-inflammatory cytokines such as, TNF- α, iNOS and Cox-2 expression in comparison to the non-user group (twelve non-users), but the difference of IL-6 expression was not statistically significant (p > 0.05) (Fig. 4A,B).

## Discussions

Chewing of SLT is an age old habit among humans. The effects of SLT on physiological systems are also well-known. However, the present study is a comprehensive attempt, where SLT mediated systemic toxicity, physiological perturbation and cellular abnormalities have been investigated in detail. We have shown that SLT chewers have altered RBCs. Transformed membrane fluidity after cigarette smoking has also been reported in platelets^23^. We have shown that, with high magnification of scanning electron microscopy (SEM), SLT-users have changed RBC membrane with loss of their typical discoid shape. Fine “bubble-like” protrusions were present on RBC membrane surfaces in the chewers group, as can be seen in Fig. 1B. We believe that the ingredients or the metabolic derivatives of SLT perturb the cellular metabolism of the individuals leading to alteration of shape and morphology of RBC. RBC shape and conformational change have huge consequences in the context of maintaining health.

Chewing tobacco altered the cell cycle dynamics. In SLT-user group, significant enhancement in the cell percentage was observed at the G0/G1 phase (p < 0.05), in comparison to the non chewer group. The cell cycle regulates the process of cellular proliferation and growth. It is known that cyclins and CDK are two crucial regulatory molecules, which determine progress of cells through the cell cycle. The regulatory unit cyclin can bind to the activated catalytic partner CDK, forming the functional cyclin–CDK complexes. For instance, the G0/G1 transition is regulated by cyclin-D-CDK-4 and cyclinD-CDK-6; G1/S transition is regulated by cyclin-E-CDK-2. The different cyclin–CDK complexes display distinct physiological functions. In the current study, there was an obvious percentage increase of G0/G1-phase with a concomitant percentage reduction of G1-phase in SLT-user group (Fig. 2A). In addition, up-regulated expressions of CDK-4 and CDK-6 and down-regulated expressions of cyclin-E and CDK-2 were both detected in SLT-user group (Fig. 2C), which contributed to the blockage of G0/G1 transition^24^. According to the result, it could be inferred that SLT induced cell cycle arrest at G1- phase by down-regulating the expressions of CDK-2 and cyclin-E, which blocked the progression from G1 to S phase of the cell cycle^22^,^25^. This subsequently leads to the stimulation of expression of p53 and p21. p53/p21 pathway is crucial in the cellular response to DNA damage^26^. In response to persistent DNA damage, the expression of p53 and p21 was enhanced, trying to halt cell proliferation and keeping time for repairing of damaged DNA. It is well known that DNA damage induces p53 and p21, ultimately leads to cell cycle arrest^27^. Moreover, we observed that chewing tobacco increased the expression of p53 and p21 in PBMC, which suggested that the prolonged habit of tobacco chewing may be the cause for cell cycle arrest in the G0/G1 phase. SLT and its components were responsible for various cellular activities, including perturbing the pro- and anti-apoptotic pathways. In our experimental set up, we found increased expression of Bax, a pro-apoptotic protein in chewers compared to the non-chewers. However, non-chewers showed increased level of expression of anti-apoptotic protein, Bcl-2. The chewing of tobacco could lead to interruption of cell cycle progression through the p53/p21 signalling axis and induced cell cycle arrest leading to subsequent apoptosis through Bcl-2 and Bax disequilibrium. We found higher TNF-α (p < 0.05) and IL-6 (p > 0.05) expressions in the user group. IL-6 is critical as an anti-inflammatory mediator by inhibition of other cytokines such as IL-1, IL-10 and TNF-α^28^. In inflammatory processes, the inducible isoform of cyclooxygenase (COX-2) is expressed in many cells, including fibroblasts and macrophages, accounting for the release of large quantities of pro-inflammatory prostaglandins at the site of inflammation^29^. The NOS and COX systems are often present together, share a number of similarities and mediate fundamental roles in pathophysiological conditions^30^. Increasing evidences suggest that there is considerable ‘cross-talk’ between COX and NOS. *In vivo* studies revealed that the regulation of COX by NO is a powerful mechanism that is used to magnify the course of the inflammatory response^31^. In our study, we found significant difference in iNOS and COX-2 expression in the user group, which is a peripheral toxicological effect of long term habit of chewing tobacco.

Lipid peroxidation (LPO) products diffuse from the inflammatory site and can be measured in the blood^32^. Nicotine and TSNA induced toxicity may cause the production of reactive oxygen species (ROS) which in turn, cause the generation of LPO. Erythrocyte membrane from SLT-user group showed significantly higher (p < 0.05) level of MDA formation which amplified the levels of TBARS as compared to normal subjects. LPO has been reported to be directly proportional to oxidative stress, in which the efficacy of defence mechanism is weakened and higher LPO also is an indicator of membrane damage. Thus, the phenomenon of peroxidation in PBMC and RBC membrane, in SLT-users is prevalent in certain cancer patients’ viz. gastric, laryngeal, oral cancer and malignant lymphoma^33^,^34^,^35^,^36^ etc. Biochemical evidences have demonstrated that reduced glutathione plays a central role in cellular defence against ROS^37^. The tri-peptide glutathione (glutamyl-cysteinyl-glycine) plays a very crucial role in protecting cells from free radicals and peroxides. Chewers showed significantly lower (p < 0.05) level of GSH content in PBMC. The reduction of GSH content in SLT-user may pose to be the direct or indirect effect of nicotine and TSNA induced ROS or direct effect of nicotine to GSH content of PBMC and RBC membrane. We found the change of catalase and SOD activities were not statistically significant both in case of PBMC and RBC membrane. Though the difference is not statistically significant at 0.05 level, there was an apparent trend towards significance for the level of TBARS of PBMC (p = 0.09) (Table 2) and GSH of RBC membrane (p = 0.07) (Table 3). We would have obtained significant difference with larger sample size. These observations reflect insufficient antioxidant status in PBMC and erythrocytes of chewers.

**Figure 5:**
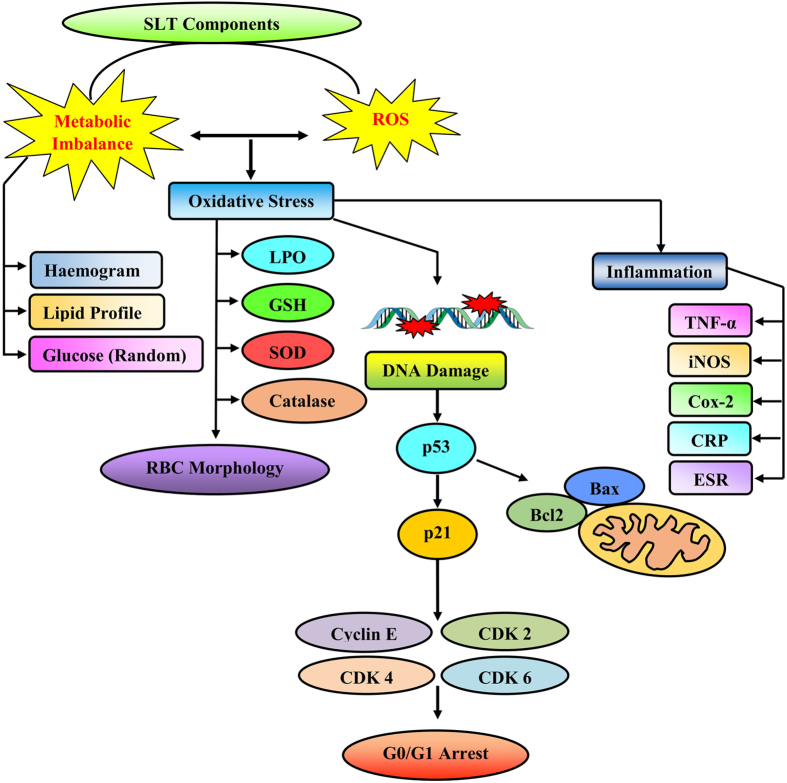
Schematic illustration of possible mechanism of action of SLT components mediated damage.

Results obtained in this study, are in accordance with other published reports on the impact of smoking on several markers of systemic inflammation^38^,^39^. We found significantly higher level of expression for markers of systemic inflammation like CRP and ESR in the SLT-user group compared to the non-user group. CRP is a marker of inflammation that predict incidents like myocardial infarction, stroke, peripheral arterial disease, sudden cardiac death among healthy individuals with no history of cardiovascular disease, and recurrent events with death in patients with acute or stable coronary syndromes^30^. Therefore, enhancement of CRP in chewers is one of the indications of inflammation in the body. This may cause cardiovascular disorders or other inflammatory manifestations^40^. A higher count of leucocytes and neutrophils was found in SLT-user group, a finding which is consistent with some previous studies on smokers^41^,^42^. Regular SLT-users take comparable amount of nicotine as cigarette smokers^15^. However, nicotine or other associated compounds are absorbed slowly. This situation remains as persistent threat for regular users. Thus the finding of dyslipidemia in SLT-users is an additional risk. Nicotine has a high half-life and remains in blood for a longer time^43^,^44^. It is quite obvious to explain that these manifestations of toxicological challenge like RBC membrane alteration, apoptotic conditions were caused by the chemicals of SLT. Nicotine in general, induces release of catecholamine. The later for its prolonged presence, triggers inflammation and contributory reason for the higher leukocyte count among the chewers. Lower lymphocyte level and higher value of ESR in the chewer group may be due to the long term habit of using SLT. Nicotine is considered as a vasoconstrictor^45^, it narrows blood vessels, slows down blood flow and reduces the amount of oxygen reaching the tissues. The earlier events cause local damage of tissues, a process causing an elevation of blood glucose and cholesterol. Nicotine can also increase insulin resistance by making cells less responsive to insulin. However, less is known about smokeless tobacco, although the regular oral use of snuff is associated with blood levels of nicotine similar to those observed in cigarette smokers^46^. We explain that increased sugar and dyslipidemic conditions were developed either to combat the systemic stress or it is the direct effect of SLT components in blood for chronic period. Published literatures reported that oral use of moist snuff is associated with an increased risk of cardiovascular death^15^. Therefore, high level of blood sugar and cholesterol in chewers may have the effect of nicotine on their body system, which may enhance the risk of myocardial infarction. Elevated levels of serum cholesterol and TG are major risk factor for coronary artery disease. Cholesterol is an essential constituent of lipoprotein fractions like LDL-C, HDL-C and VLDL-C. LDL-C is the “bad” cholesterol because on its elevation, it can promote atherosclerotic heart disease. In our study, we found chewers have elevated levels of LDL-C and VLDL-C which may cause the development of coronary artery diseases. High platelet count in SLT-user may block the blood vessels augmenting the risk of cardiovascular diseases. Serum creatinine is an important indicator of renal health and a by-product of muscle metabolism excreted unchanged by the kidneys. Normal level of serum urea and creatinine in the studied groups indicated that kidney functions of both the groups were normal. Therefore, from the studied group, it may be conferred that, SLT remains a threat locally in the oral cavity and following its absorption; it modulates the metabolic pattern in a robust way, escalate the risk of systemic inflammation and promotes the risk of coronary artery disease, cardiovascular disease and dyslipidemia (Fig. 5). This is a comprehensive evidence of systemic toxicity, apoptosis, cell cycle arrest, metabolic disorder and RBC morphology modulation after smokeless tobacco chewing.

## Materials and Methods

### Chemicals

Histopaque-1077, Trichloroacetic acid (TCA), Thiobarbituric acid (TBA), 5, 5′-dithio-bis (2-nitro benzoic acid) (DTNB) were purchased from Sigma Aldrich (St. Louis, MO, USA). Antibodies (p53, p21, Bax, Bcl-2, IL-6, TNF-α, iNOS, COX-2, Cyclin-E, CDK-2, CDK-4 and CDK-6) were purchased from Cell Signalling Technology (Danvers, MA, USA). RPMI Medium-1640 was purchased from Life Technologies (Grand Island, USA). Ethidium bromide (EtBr), hydrogen peroxide (H_2_O_2_), ethanol and all other fine chemicals were procured from Merck (Germany).

### Ethical approval

We obtained ethical approval from the Institutional Human Ethics Committee, Department of Physiology of University of Calcutta (Ref. No. IHEC/SD/P02/11 dated 13.04.2011). An information sheet describing the rationale of the study and individuals’ rights was handed to the participants to read. For individuals with inadequate literacy, the information sheet was read out by the interviewers. Written informed consent was then obtained from each person. Thumb impression was obtained from those who were unable to sign the consent form.

### Study area and data collection

Four different camps were organised at two Government offices in Kolkata, India. Total 91 (N = 91) Government employees were included in this study who reside in this city for the last 20 years. The entire studied population was subdivided into two groups: i) SLT-users, individuals consume only SLT, not any other form of tobacco and ii) Non-users, individuals do not consume any form of tobacco. The mean age of SLT-user and non-user was (48.87 ± 7.002) and (48.30 ± 8.74) year respectively. The baseline information for the category of SLT-users was that individuals used SLT products habitually, at least >20 times per week for the last 6 months, while occasional users and one time users were placed in the category of non-users. Only non-smokers were included in this study. 5 ml venous blood was collected from all the individuals. A specific diet chart was supplied to all the subjects before two months of blood collection. During this period they were under continuous monitoring of the health issues, diet condition and related paraphernalia. A written consent form was collected from the participants; information was gathered about the history of SLT consumption, knowledge and attitudes concerning the habit of SLT consumption. Socio-demographic information was collected by an interviewer who performed the face-to-face interview, with the information on: age, educational qualification, marital status, income, occupational status and religion. All experiments were performed in accordance with the approved guidelines and regulations of Human Ethics committee and the Institutional Bio-Safety Committee of University of Calcutta.

### Analysis of clinical parameters

A spectrum of clinical and haematological parameters like CRP, ESR, glucose (random), urea, creatinine, haemoglobin, total lipid profile were analysed from all studied individuals of the SLT-user group (N = 47) and non-user group (N = 44) from their blood using autoanalyzer (Purechem Ltd, Ireland).

### Determination of LPO

The formation of TBARS in the homogenate was estimated using standard protocol^47^ as the marker of lipid peroxidation. Briefly, the cell fractions (lysate and RBC membrane) were mixed with 15% TCA, 0.375% TBA and 5N HCl followed by incubation at 95 °C for 15 min. The mixture was cooled, centrifuged and the absorbance of supernatant was measured at 535 nm against appropriate blank.

### Determination of SOD activity

SOD was determined using modified pyrogallol auto-oxidation method^48^. In brief, cell fractions were added to 62.5 mM tris-cacodylic acid buffer followed by 4 mM pyrogallol. The auto-oxidation of pyrogallol was monitored at 420 nm, followed by the estimation of the absorbance of the test samples at specific time intervals.

### Determination of GSH activity

Cell fractions were treated with 0.1 ml of 25% TCA and the resulting precipitate was pelleted by centrifugation at 3,900 × *g* for 10 min. Free endogenous sulfydryl group was assayed in a total 3 ml volume mixture (2 ml of 0.5 mM DTNB prepared in 0.2 M phosphate buffer (pH 8.0), with 1 ml of the supernatant). The GSH reacted with DTNB and formed a yellow complex with DTNB. The absorbance was read at 412 nm^48^.

### Determination of catalase activity

Absorbance of H_2_O_2_ was taken at 240 nm followed by evaluation of the decrease in absorbance in the cell lysate indicating the elimination of H_2_O_2_ by the action of catalase. Cell fractions were mixed with 50 mM potassium phosphate buffer (pH 7.0) and 30 mM H_2_O_2_ in a total reaction volume of 1.0 ml. The reaction was then carried out at 20 °C and only the initial linear rate of absorbance was used to estimate the catalase activity^48^.

### Whole blood smear preparation for scanning electron microscopy

RBC morphology was studied on whole blood smears using scanning electron microscope following slightly modified the protocol of Pleterious *et al.*^49^. Whole blood was diluted 1:1 with phosphate buffered saline (PBS, pH 7.4) and placed it at 37 °C for 10 min. Samples were centrifuged three times at 1,500 rpm for 5 min before they were fixed in 2.5% glutaraldehyde in Dulbecco’s phosphate buffered saline (DPBS) solution with pH of 7.4 for 60 min. The samples were rinsed and washed with phosphate buffer three times for 5 min before being fixed with 1% osmium tetra-oxide (OsO_4_). This was followed by another three times rinsing with PBS for 5 min each time, followed by a serial dehydration in 30%, 50%, 70%, 90% and three times with 100% ethanol. A smear of the preparation was prepared on a glass cover slip, dried, mounted and coated with platinum. A ZEISS EVO-MA 10 (Jena, Germany) system was used to study the surface morphology of erythrocytes.

### Isolation of PBMC

PBMC were immediately isolated from fresh whole blood by density gradient centrifugation according to the method of Boyun^50^ using equal volume of blood and Ficoll (Histo-paque 1077). Briefly, 5 ml blood was layered carefully over equal volume of Histopaque 1077 and subjected to centrifugation for 30 min at 400 × *g*. PBMC were collected from the buffy layer formed at the plasma– Histopaque 1077 interface and the pellet was re-suspended in PBS (50 mM, pH 7.4). This process was repeated twice or thrice to remove the extraneous platelets. The viable cell number was determined using the trypan blue staining technique.

### Isolation of RBC membrane

Freshly drawn blood was used for membrane preparation. The RBC membrane was prepared using the protocol of Dodge *et al.*^51^ after minor modification. Briefly, hypotonic phosphate buffer was added to the suspension of erythrocyte and centrifuged at 25,000 × *g* for 40 min. Red, loosely packed membrane pellet was resuspended in hypotonic phosphate buffer and centrifuged at 20,000 × *g* for 20 min. This step was repeated five to six times more till a milky-looking membrane pellet was formed and it was re-suspended in PBS (pH 7.4).

### Cell cycle analysis

The cell cycle distribution was analyzed using flowcytometer. PBMC (1 × 10^6^) were harvested and rinsed twice with PBS. The cell pellets were fixed in 70% ice cold ethanol at 4 °C overnight. Then cells were centrifuged at 1,200 rpm for 5 min followed by 1 h incubation with 1.0 mg/ml of RNase at 37 °C. The cells were stained with 50 μg/ml PI containing 0.1% Triton X-100 and 0.02 mg/ml EDTA. The percentage of DNA content in each phase of the cell cycle was evaluated by BD FACSAria III flow cytometer (Becton Dickinson, Franklin Lakes, NJ). The data were analysed using Flow Jo Software (Version 10.0)^52^.

### Analysis of protein expression by flow cytometry

Cells were fixed in 4% paraformaldehyde in PBS (pH 7.4) for 20 min at room temperature and permeabilized in 0.1% Triton X-100 in PBS with 0.1% FBS for 5 min. After washing twice in PBS with 3% FBS, the permeabilized cells were incubated with primary antibody at 4 °C for overnight and washed twice in PBS. The cells were then incubated with FITC-conjugated or Alexa Fluor 647-conjugated goat anti-rabbit IgG as a secondary antibody for 30 min on ice and washed twice in PBS. The stained cells were acquired and analyzed using a BD FACSAria III flow cytometer (Becton Dickinson, Franklin Lakes, NJ, USA) and data were analysed with Flow Jo software^53^.

### Preparation of cell lysate of PBMC

Isolated PBMC (8 × 10^6^) was suspended in 100 μl hypotonic buffer (1.5 mM MgCl_2_, 10 mM KCl, 1 mM dithiothreitol, 10 mM HEPES, pH −7.9) containing protease inhibitor cocktail and sonicated. The suspension containing the ruptured cells was centrifuged at 13,000 × *g* for 10 min at 4 °C. The supernatant (cell homogenate) was then used for subsequent antioxidant enzyme assays and western blot analysis.

### Western blot analysis

Among the studied population thirteen SLT-users (N = 13) and twelve non-users (N = 12) were randomly selected for molecular analysis. Immunblots were performed from cell lysate to analyze the expression of different cell cycle and apoptotic markers, such as, p53, p21, Bax, Bcl-2 and from serum to measure the level of different pro-inflammatory cytokines, such as, IL-6, TNF-α, iNOS, Cox-2. GAPDH and β-actin were used as loading control respectively. Concentration of proteins was determined by the protocol of Lowry *et al.*^54^. Equal amount of protein (50 μg) was loaded on each lane followed by separation using sodium dodecyl sulphate-polyacrylamide gel electrophoresis (SDS-PAGE) and electroblotted in PVDF membrane (Millipore, Massachusetts, USA). The membrane was blocked for 2.0 h at 37 °C with 5% bovine serum albumin (BSA) solution. Then the membrane was incubated with anti-rabbit polyclonal antibody (1:1000) for overnight at 4 °C, followed by incubation with alkaline conjugated anti rabbit antibody (1:2500) for 2.0 h. After washing, the membrane was developed using a NBT-BCIP chromogenic detection system^48^. For each result three independent set of western blot experiments were performed for different subjects. The current result is the representative of three western blot data. All western blots were performed under the same experimental conditions.

### Statistical analysis

For biochemical analysis, summary statistics were analysed using SPSS 20.0 and the mean ± standard deviation (SD) were tabulated in Tables 1, 2, 3. Linear regression was performed keeping all the biological parameters as response variable and age, sex and SLT habit status as explanatory variables. For each biochemical parameter, an independent regression analysis was performed. In the analyses, age and sex adjusted p-values with respect to the SLT habit status were tabulated using one-tailed two-sample t-test. Significant evidence of the effect of SLT habit status on the different biochemical parameters was inferred based on the adjusted p- values. In other words, we identified those biological parameters for which SLT-users showed significant differences compared to non-tobacco users. For cell cycle analysis all data were presented as mean ± standard error of mean (SEM). Densitometric analyses of the western blots were done using ImageJ software and the data are represented as mean ±  SEM. The significance of differences between the means of SLT-user and non-user groups was determined by one-tailed two-sample t-test using Origin Pro 8.0 software. In all cases p-value <0.05 was considered as significant between two test groups.

## Additional Information

**How to cite this article**: Biswas, S. *et al.* Smokeless tobacco consumption impedes metabolic, cellular, apoptotic and systemic stress pattern: A study on Government employees in Kolkata, India. *Sci. Rep.* **5**, 18284; doi: 10.1038/srep18284 (2015).

## Figures and Tables

**Table 1:** Key experimental parameters.

Parameters	SLT user	Non user	Adjusted p value
CRP (mg/l)	0.66 ± 0.46	0.32 ± 0.30	0.001*
Glucose (Random) (mg/dl)	2.05 ± 0.12	1.97 ± 0.07	0.001*
Urea (mg/dl)	1.34 ± 0.08	1.31 ± 0.09	0.08
Creatinine (mg/dl)	0.99 ± 0.08	0.98 ± 0.08	0.25
Serum Cholesterol (mg/dl)	204.23 ± 35.24	185.48 ± 38.03	0.008*
TG (mg/dl)	158.83 ± 57.73	130.80 ± 63.45	0.01*
HDL-C (mg/dl)	1.63 ± 0.04	1.62 ± 0.02	0.02*
LDL-C(mg/dl)	128.26 ± 28.86	117.93 ± 31.05	0.05*
VLDL-C(mg/dl)	1.48 ± 0.18	1.37 ± 0.19	0.002*
Haemoglobin (gm%)	13.27 ± 1.53	13.58 ± 1.28	0.85
Erythrocytic Count (millions/cu.mm)	4.85 ± 0.49	4.94 ± 0.39	0.82
Leucocytic Count (cu.mm)	8217.02 ± 1859.911	7831.82 ± 1821.036	0.164
Neutrophil Count (%)	64.79 ± 6.12	62.07 ± 6.55	0.02*
Lymphocyte Count (%)	32.21 ± 5.82	34.64 ± 6.25	0.97
Monocyte Count (%)	0.03 ± 0.09	0.09 ± 0.14	0.006*
Eosinophil Count (%)	0.20 ± 0.24	0.23 ± 0.24	0.35
Platelet Count (lakhs/cu.mm)	0.42 ± 0.08	0.40 ± 0.09	0.1
ESR (mm)	1.35 ± 0.37	1.19 ± 0.27	0.01*
PCV (%)	39.44 ± 4.67	40.46 ± 3.88	0.12
MCV (fl)	80.97 ± 7.30	81.63 ± 4.80	0.3
MCH (pg)	27.31 ± 2.64	26.83 ± 4.30	0.26
MCHC (%)	33.72 ± 0.70	33.60 ± 0.67	0.21

Values indicate Mean  ±  SD *Adjusted p value <0.05.

**Table 2:** Antioxidant parameters from PBMC.

Parameters (PBMC)	SLT user	Non user	Adjusted p value
TBARS (nmoles/mg of protein)	3.70 ± 0.51	3.56 ± 0.40	0.09
GSH (nmoles/mg of protein)	42.72 ± 26.40	53.25 ± 28.03	0.03*
SOD (U/g of protein)	1.26 ± 0.34	1.1 ± 0.33	0.81
Catalase (mmoles of H_2_O_2_ reduced/mg of protein)	2.08 ± 0.56	2.10 ± 0.55	0.44

Values indicate Mean  ±  SD *Adjusted p value <0.05.

**Table 3:** Antioxidant parameters from RBC membrane.

Parameters (RBC Membrane)	SLT user	Non user	Adjusted p value
TBARS (μmoles/mg of protein)	15.16 ± 11.73	8.14 ± 9.71	0.001*
GSH (nmoles/mg of protein)	366.47 ± 137.98	415.41 ± 173.71	0.07
SOD (U/g of protein)	1.26 ± 0.37	1.04 ± 0.26	0.99
Catalase (mmoles of H_2_O_2_ reduced/mg of protein)	1.75 ± 0.49	1.55 ± 0.57	0.96

Values indicate Mean ± SD *Adjusted p value <0.05.
